# Emerging role of GLP-1 mono, dual, and triple agonists in the management of MASH-related fibrosis

**DOI:** 10.1016/j.xcrm.2026.103023

**Published:** 2026-09-04

**Authors:** Federica Tavaglione, Rohit Loomba

**Affiliations:** 1 MASLD Research Center, Division of Gastroenterology and Hepatology, University of California, San Diego, La Jolla, CA, USA; 2 School of Public Health, University of California, San Diego, La Jolla, CA, USA

**Keywords:** MASLD, NAFLD, incretins, glucagon, GIP

## Abstract

Recent therapeutic advances have resulted in US Food and Drug Administration (FDA) approval of the first pharmacological agents—resmetirom and semaglutide—for advanced metabolic dysfunction-associated steatohepatitis (MASH) without cirrhosis, reshaping the therapeutic landscape of the disease. Within this evolving framework, incretin-based pharmacotherapies—including glucagon-like peptide-1 (GLP-1) receptor agonists and their dual and triple combinations with glucose-dependent insulinotropic peptide (GIP) and/or glucagon receptor agonists—have emerged as promising options, particularly for individuals with coexisting obesity or type 2 diabetes. These agents exert pleiotropic effects across multiple organs, modulating glucose and lipid metabolism, while providing cardiovascular and renal benefits. Despite these advances, key challenges remain regarding treatment duration, tolerability, and interindividual variability in therapeutic response. This review summarizes the emerging role of GLP-1 mono, dual, and triple agonists in MASH-related fibrosis, focusing on their mechanisms of action and evidence from phase 2 and phase 3 clinical trials with histology-assessed hepatic endpoints.

## Graphical abstract

**Figure:**
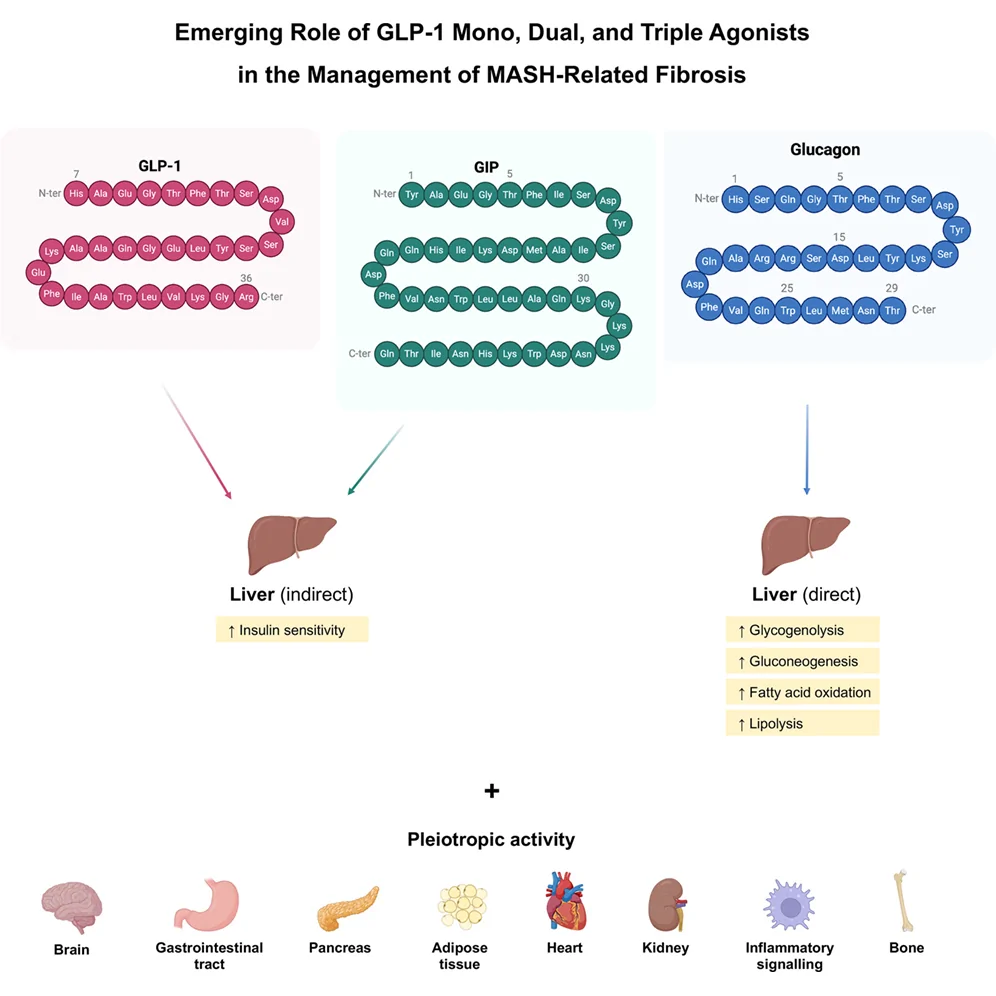

## Teaser

Tavaglione and Loomba review the emerging role of GLP-1 mono, dual, and triple agonists in MASH-related fibrosis. These therapies target multiple metabolic and inflammatory pathways and show promising hepatic histological benefits, highlighting their potential to reshape MASH-related fibrosis treatment, while underscoring remaining challenges in treatment duration, tolerability, and individual response.

## Introduction

Metabolic dysfunction-associated steatotic liver disease (MASLD) is a leading cause of chronic liver disease worldwide and a major public health concern, affecting up to 38% of the global population and projected to reach 55% by 2040.^1^^,^^2^^,^^3^ MASLD is a heterogeneous condition primarily driven by obesity and type 2 diabetes that encompasses a dynamic spectrum of conditions, from isolated hepatic fat accumulation to inflammation, that is, metabolic dysfunction-associated steatohepatitis (MASH), and fibrosis, ultimately progressing to cirrhosis, hepatocellular carcinoma, and end-stage liver disease.^4^^,^^5^

Hepatic fibrosis is the strongest predictor of liver-related complications and overall mortality in individuals with MASLD, particularly among those with MASH and fibrosis stage ≥ 2, a condition referred to as progressive or at-risk MASH.^6^^,^^7^^,^^8^ Given the central role of fibrosis and disease activity in determining prognosis, regulatory agencies such as US Food and Drug Administration (FDA) and European Medicines Agency (EMA) currently consider two histological endpoints suitable for accelerated drug approval in the context of MASH clinical trials: (1) resolution of MASH without worsening of fibrosis, and (2) improvement in fibrosis by at least one stage without worsening of MASH.^9^ These endpoints serve as critical treatment objectives and are commonly employed as surrogate indicators of long-term clinical benefit. Additional endpoints commonly assessed in early-phase trials include non-invasive biomarkers such as alanine transaminase (ALT) and aspartate transaminase (AST), liver fat content measured by magnetic resonance imaging-proton density fat fraction (MRI-PDFF), and liver stiffness evaluated using elastography.^2^

Over the past decade, a substantial body of clinical trials have been conducted in the field of MASH aimed at evaluating the safety and efficacy of novel therapeutic agents.^10^ These efforts have culminated in the recent FDA approval of two pharmacological agents—resmetirom (March 2024)^11^ and semaglutide (August 2025)^12^—for the treatment of MASH with significance to advanced fibrosis. In this evolving therapeutic landscape, incretin-based pharmacotherapies—including glucagon-like peptide-1 (GLP-1) receptor agonists, alone or in combination with glucose-dependent insulinotropic peptide (GIP) and/or glucagon receptor agonists—are increasingly emerging as promising therapeutic options for MASH, particularly in individuals with coexisting obesity or type 2 diabetes.^13^^,^^14^^,^^15^^,^^16^ In this review, we discuss the emerging role of GLP-1 mono, dual, and triple agonists in the management of MASH-related fibrosis, highlighting their pathophysiology, mechanisms of action, and the current evidence from phase 2 and 3 clinical trials.

### Physiology and mechanisms of action

#### Incretin hormones

**Figure 1:**
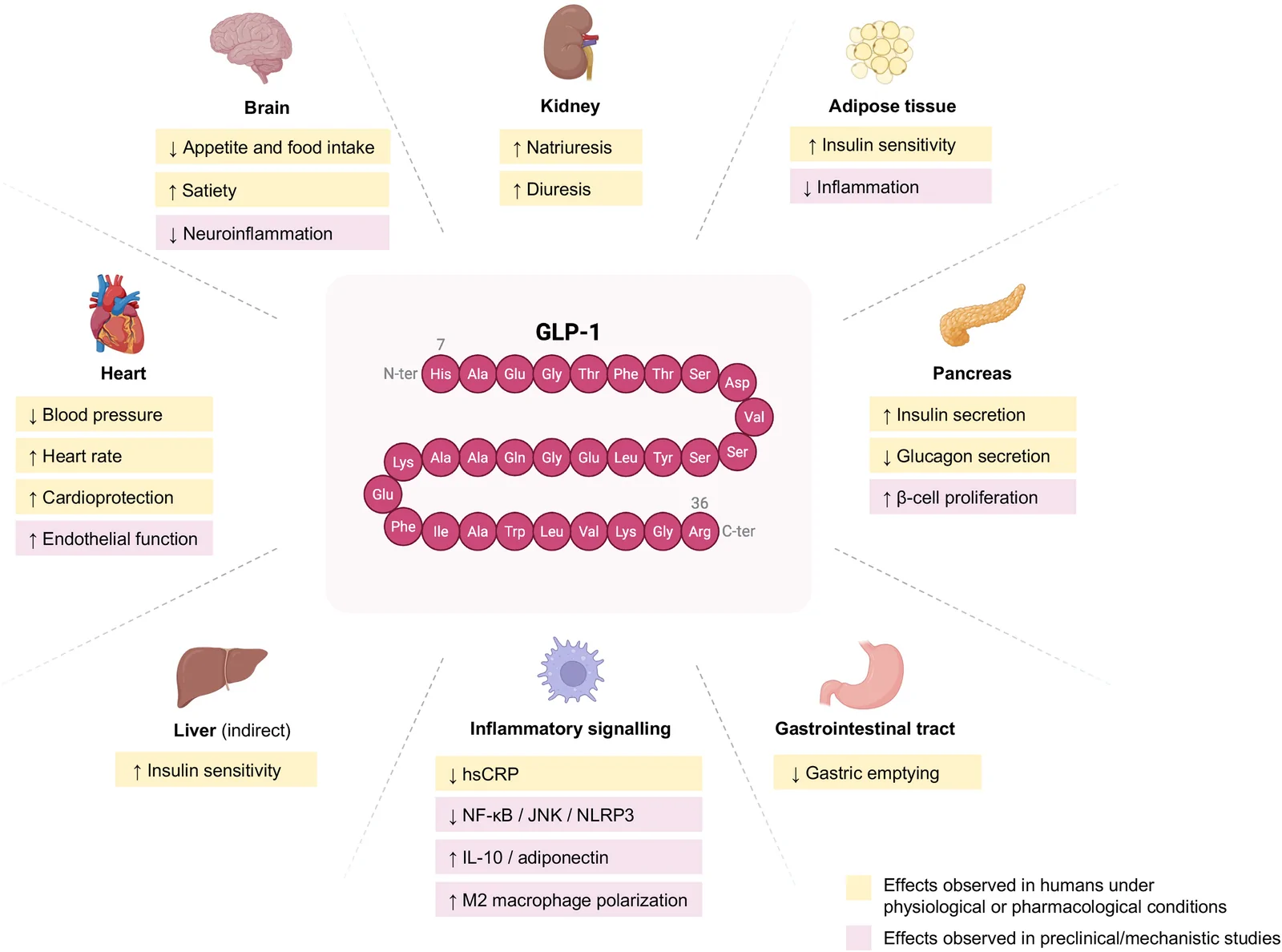
Effects of GLP-1 receptor agonism This figure was created using BioRender (https://biorender.com/). Abbreviation: GLP-1, glucagon-like peptide-1.

**Figure 2:**
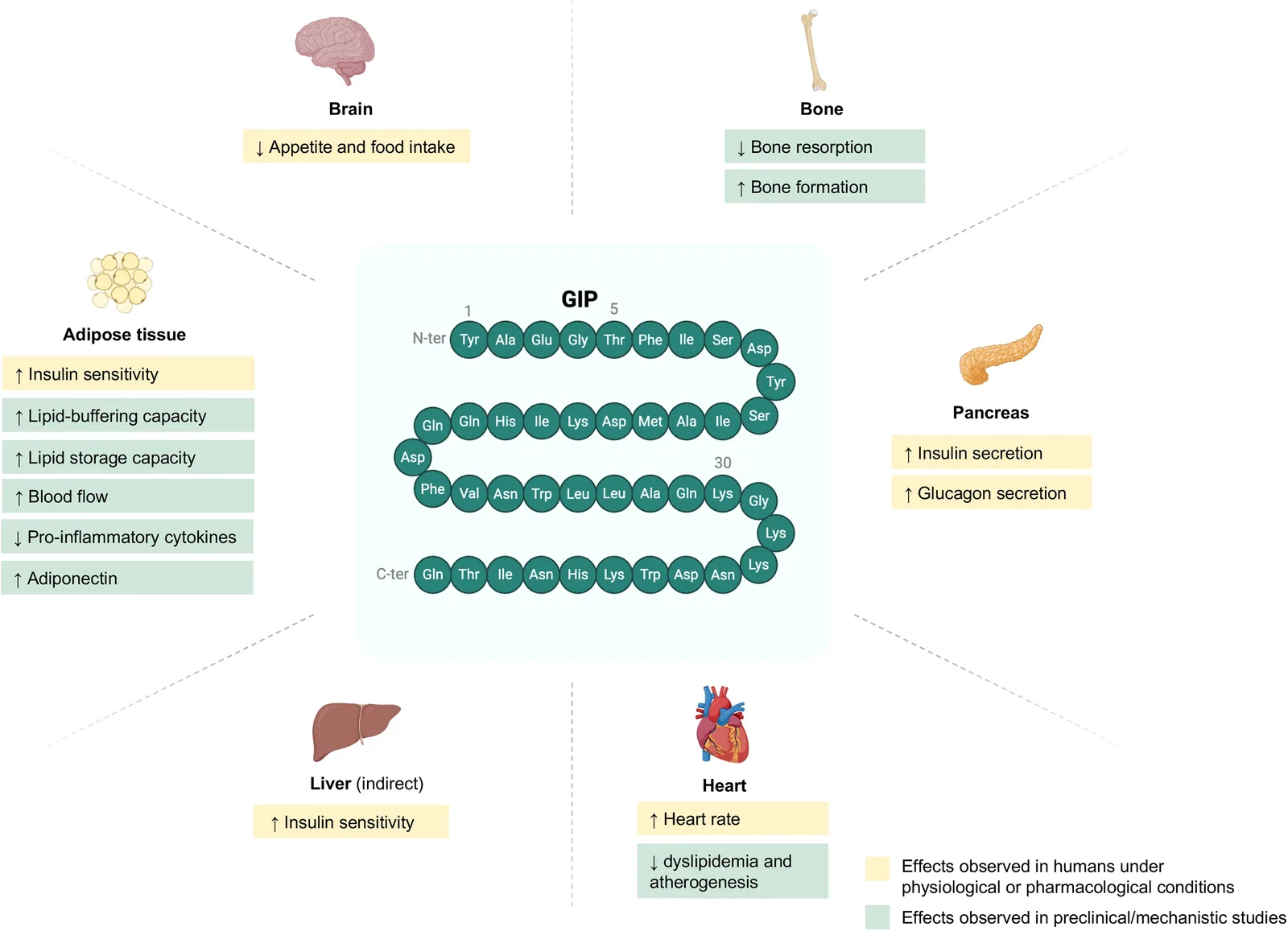
Effects of GIP receptor agonism This figure was created using BioRender (https://biorender.com/). Abbreviation: GIP, glucose-dependent insulinotropic peptide.

Incretin hormones, including GLP-1 and GIP, are gut-derived polypeptides released in response to oral nutrient intake that enhance insulin secretion from the pancreas in a glucose-dependent manner. Additionally, these hormones exert pleiotropic effects on multiple tissues, including in the gastrointestinal tract and nervous system, influencing glucose and lipid metabolism, promoting weight loss, and improving cardiovascular and kidney health^2^^,^^7^^,^^17^ (Figures 1 and 2).

GLP-1 is a short-acting incretin hormone (half-life < 2 min) secreted by intestinal enteroendocrine L cells following the ingestion of fat- and carbohydrate-rich meals.^18^ GLP-1 receptor agonism confers a wide range of metabolic benefits, including glucose-dependent stimulation of insulin secretion, suppression of glucagon release, promotion of β cell proliferation, and enhancement of thermogenesis in brown adipose tissue.^19^ In addition, GLP-1 receptor agonism exerts central effects within the hypothalamus to reduce appetite and peripheral actions in the gastrointestinal tract to slow gastric emptying.^13^ GLP-1 receptors are not expressed on the hepatocytes; therefore, the hepatic benefits of GLP-1-based therapies were largely considered indirect and attributed primarily to weight reduction and blockade of free fatty acids trafficking to the hepatocytes.^2^^,^^20^^,^^21^^,^^22^ However, there is emerging evidence supporting a weight-independent effect of semaglutide, including immunomodulatory actions across multiple inflammatory pathways relevant to MASH pathogenesis.^23^^,^^24^^,^^25^^,^^26^ In preclinical models, GLP-1 receptor agonists suppressed key inflammatory signaling pathways, including NF-κB, JNK, and the NLRP3 inflammasome, resulting in reduced expression and production of pro-inflammatory cytokines such as TNF, IL-1β, and IL-6.^27^^,^^28^ Consistently, studies in murine and cellular models have demonstrated the attenuation of endotoxin-induced TNF signaling, increased production of anti-inflammatory mediators such as IL-10 and adiponectin, and promotion of macrophage polarization toward an anti-inflammatory M2 subtype.^29^^,^^30^^,^^31^ In endothelial cells, GLP-1 receptor agonists activated AMPK signaling and suppressed the expression of inflammatory adhesion molecules such as VCAM-1 and E-selectin, thereby reducing monocyte activation and vascular inflammation.^32^ GLP-1 receptor agonism has also been shown to modulate the systemic inflammatory tone via central nervous system pathways, contributing to the suppression of systemic TNF through α1-adrenergic, δ-opioid, and κ-opioid receptor signaling.^33^ In addition, semaglutide reduced adipose tissue inflammation in murine models, supporting broader anti-inflammatory and cardiometabolic effects.^34^ In line with these findings from preclinical studies, *in vivo* studies have demonstrated that multiple GLP-1 receptor agonists reduce the serum levels of high-sensitivity C-reactive protein (hsCRP), an acute-phase reactant produced under the control of pro-inflammatory cytokines such as TNF, IL-1, and IL-6.^22^^,^^25^ Furthermore, a recent study integrating clinical data from patients treated with semaglutide and serum proteomic analyses identified 72 MASH resolution-associated circulating proteins, most of which were linked to the metabolic, inflammatory, and fibrotic pathways, thereby further supporting its weight-independent anti-inflammatory and antifibrotic effects.^23^ Lastly, a secondary analysis of the phase 3 ESSENCE trial demonstrated that patients with MASH receiving semaglutide experienced improvements in liver health-related parameters, even with modest weight loss, and that the effects of semaglutide on MASH-related histologic endpoints were only partially mediated by body weight reduction.^24^ GLP-1 receptors are also expressed across multiple neural and vascular cell types, including astrocytes and microglia, within brain regions that are particularly vulnerable to Alzheimer disease pathology. Recent studies have further provided evidence for the role of GLP-1 receptor agonists in attenuating neuroinflammatory changes in preclinical models, supporting their potential as candidate therapeutic agents for Alzheimer disease.^35^^,^^36^ However, the first two double-blind, placebo-controlled, phase 3 trials to assess the efficacy and safety of semaglutide in Alzheimer disease did not slow disease progression.^37^

GIP is a short-acting incretin hormone (half-life = 7 min) released by intestinal enteroendocrine K cells in response to fat- and carbohydrate-rich meals. GIP stimulates insulin secretion from the pancreas, although its insulinotropic effect is less potent than that of GLP-1.^7^^,^^38^ As with GLP-1, GIP receptor expression is limited in the liver and is not considered a direct site of action, yet GIP agonism exerts beneficial hepatic effects by improving insulin sensitivity, reducing inflammation, and modulating lipid metabolism.^7^^,^^17^ Notably, GIP plays a central role in adipose tissue biology that extends beyond its classical incretin function. Unlike GLP-1, the GIP receptor is expressed in adipocytes,^39^ and preclinical studies in animal models suggest that GIP receptor agonism enhances lipid buffering by promoting dietary triglyceride uptake, improving triglyceride clearance, and increasing lipoprotein lipase activity and triglyceride storage in white adipose tissue.^40^^,^^41^^,^^42^ Consistent with these findings, experimental studies in humans have demonstrated that GIP promotes triglyceride storage in white adipose tissue, increases adipose tissue perfusion, and reduces circulating free fatty acids, although these effects may be attenuated in individuals with type 2 diabetes.^43^^,^^44^^,^^45^^,^^46^ Importantly, beyond facilitating acute dietary triglyceride clearance, GIP may promote long-term triglyceride storage by supporting healthy expansion of white adipose tissue, including stimulation of pre-adipocyte differentiation (*de novo* adipogenesis) and increasing adipocyte number rather than size.^47^^,^^48^ These lipid-buffering and -storage functions reduce lipid spillover and ectopic fat accumulation in tissues such as the liver, heart, pancreas, and skeletal muscle, thereby limiting lipotoxicity and supporting systemic metabolic homeostasis—processes that are highly relevant to MASH pathogenesis and progression.^49^ Additional mechanistic and clinical studies are needed to further validate this hypothesis. Beyond metabolic effects, preclinical studies have shown that GIP receptor signaling ameliorates adipose tissue inflammation, as indicated by reduced secretion of pro-inflammatory cytokines and chemokines and increased release of insulin-sensitizing adipokines.^50^^,^^51^ Furthermore, GIP receptor agonism inhibits bone resorption (breakdown by osteoclasts), while potentially stimulating bone formation.^52^^,^^53^^,^^54^^,^^55^ Finally, GIP receptor agonism improved dyslipidemia and atherosclerosis in mouse models.^56^

#### Glucagon

**Figure 3:**
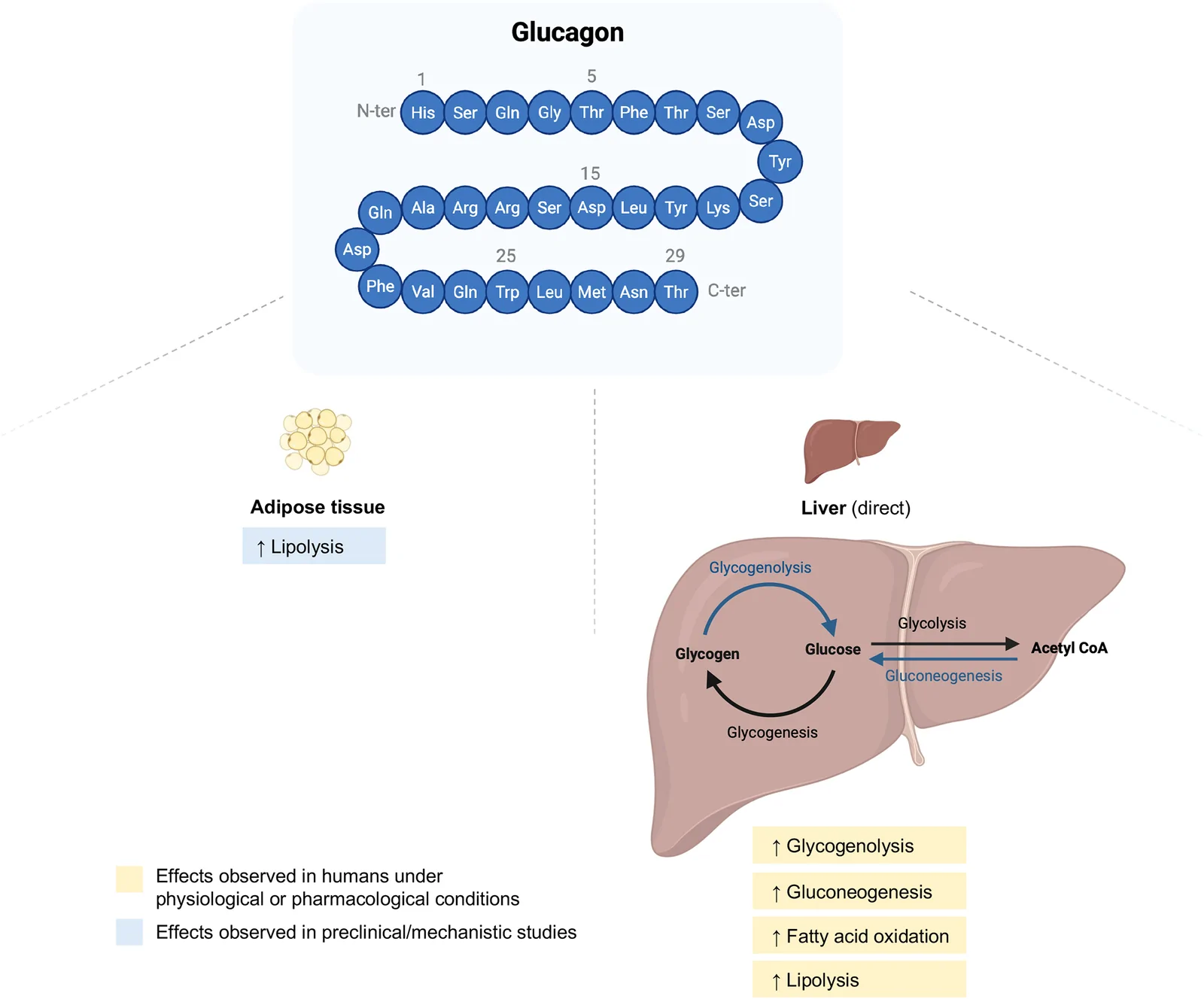
Effects of glucagon receptor agonism This figure was created using BioRender (https://biorender.com/).

Glucagon is a peptide hormone secreted by α cells of the endocrine pancreas that primarily act on the liver to maintain glucose homeostasis by stimulating glycogenolysis and gluconeogenesis. Additionally, glucagon receptor activation promotes hepatic fatty acid oxidation and lipolysis, facilitating liver triglyceride mobilization^2^^,^^57^^,^^58^ (Figure 3). Importantly, although glucagon is a potent lipolytic hormone in rodents,^59^^,^^60^^,^^61^^,^^62^^,^^63^ current evidence indicates that glucagon signaling does not directly stimulate adipose tissue lipolysis in humans, likely due to low glucagon receptor expression in human white adipose tissue.^64^^,^^65^ Consequently, the metabolic benefits of glucagon receptor agonism in humans appear to be predominantly liver centric, rather than being mediated through the direct stimulation of adipose tissue lipolysis. In particular, leveraging this liver-centric activity, the addition of glucagon receptor agonism to GLP-1 receptor agonists induces a fasting-like metabolic state that enhances hepatic lipid mobilization and reduces liver fat independent of weight loss, while incretin-mediated effects preserve glycemic control and mitigate glucagon-induced hyperglycemia.^57^^,^^66^^,^^67^ In this context, treatment with survodutide, a dual glucagon and GLP-1 receptor agonist, was associated with HbA1c reductions of up to 0.8%, indicating that the glycemic benefits of GLP-1 receptor agonism are maintained despite concomitant glucagon receptor activation.^68^

## Evidence from MASH clinical trials with histology-assessed hepatic endpoints

The following section details phase 2 and phase 3 MASH clinical trials evaluating GLP-1 receptor agonists, as well as dual or triple agonists targeting glucagon, GIP, and GLP-1 receptors, with hepatic endpoints assessed by histology. A summary of these findings is displayed in Table 1.

**Table 1:** Overview of phase 2 and phase 3 MASH clinical trials evaluating GLP-1 receptor agonists and co-agonists with biopsy-assessed liver endpoints

Drug	Study design	Study population	Duration	Dose	Administration	Endpoint MASH	Endpoint fibrosis
**GLP-1 Ras**							

Liraglutide	phase 2 (LEAN, NCT01237119)	*N* = 52 with MASH	48 weeks	1.8 mg vs. placebo	once daily s.c.	39% MASH resolution without worsening of fibrosis	26% improvement in fibrosis
Semaglutide	phase 2 (NCT02970942)	*N* = 320 with MASH F1-F2-F3	72 weeks	0.1, 0.2, and 0.4 mg vs. placebo.	once daily s.c.	36%–59% MASH resolution without worsening of fibrosis	32%–49% improvement in fibrosis without worsening of MASH
	phase 3 (ESSENCE, NCT04822181)	*N* = 1,197 with MASH F2-F3	240 weeks	2.4 mg vs. placebo	once weekly s.c.	62.9% MASH resolution without worsening of fibrosis (interim analysis at week 72, *n* = 800)	36.8% improvement in fibrosis without worsening of MASH (interim analysis at week 72, *n* = 800)

**GCG/GLP-1 Ras**							

Pemvidutide	phase 2 (IMPACT, NCT05989711)	*N* = 212 with MASH F2-F3	24 weeks	1.2 and 1.8 mg vs. placebo	once weekly s.c.	52%–58% MASH resolution without worsening of fibrosis	33%–36% improvement in fibrosis without worsening of MASH
Survodutide	phase 2 (NCT04771273)	*N* = 293 with MASH F1-F2-F3	48 weeks	2.4, 4.8. and 6 mg vs. placebo	once weekly s.c.	43%–62% improvement in MASH without worsening of fibrosis.	34%–36% improvement in fibrosis
	phase 3 (LIVERAGE, NCT06632444)	*N* = 1,800 with MASH F2-F3	52 weeks (part 1), 7 years (part 2)	6 mg vs. placebo	once weekly s.c.	not applicable, ongoing	not applicable, ongoing

**GIP/GLP-1 Ras**							

Tirzepatide	phase 2 (SYNERGY-NASH, NCT04166773)	*N* = 190 with MASH F2-F3	52 weeks	5, 10, and 15 mg vs. placebo	once weekly s.c.	44%–62% resolution of MASH without worsening of fibrosis.	51%–55% improvement in fibrosis without worsening of MASH

**GCG/GIP/GLP-1 Ras**							

Efocipegtrutide	phase 2 (HM-TRIA-201, NCT04505436)	*N* = 240 with MASH F1-F2-F3	52 weeks	2, 4, and 6 mg vs. placebo	once weekly s.c.	not applicable, ongoing	not applicable, ongoing

GCG, glucagon; GIP, glucose-dependent insulinotropic peptide; GLP-1, glucagon-like peptide-1; RA, receptor agonist.

### GLP-1 receptor agonists

#### Liraglutide

Liraglutide is currently approved for type 2 diabetes and obesity. In the LEAN trial (phase 2, double blind; NCT01237119), 52 adults, aged 18–70 years, with biopsy-confirmed MASH and BMI > 25 kg/m^2^, with or without diabetes, were randomized 1:1 to liraglutide 1.8 mg subcutaneously once daily (*n* = 26) or placebo (*n* = 26) for 48 weeks.^69^ Biopsies were performed at baseline and study end. MASH resolution without worsening of fibrosis occurred in 39% of the liraglutide group versus 9% of the placebo group (*p* = 0.019). Fibrosis progression was less common with liraglutide compared with placebo (9% vs. 36%; *p* = 0.04), although fibrosis improvement did not differ significantly between the groups (26% vs. 14%; *p* = 0.46). The participants on liraglutide lost 5.5% of body weight compared with 0.7% weight reduction in those on placebo (*p* = 0.003), and HbA1c declined by −5.7 mmol/mol (*p* = 0.03). Gastrointestinal events were more frequent in the liraglutide group than in the placebo group (nausea: 46% vs. 38%; diarrhea: 38% vs. 19%; constipation: 27% vs. 0%; vomiting: 19% vs. 12%), mostly mild or moderate. Treatment discontinuation due to adverse events was 8% in the liraglutide group versus 4% in the placebo group, with no reports of liver failure, hepatitis, or pancreatitis.

#### Semaglutide

Semaglutide is similarly approved for type 2 diabetes and obesity and has recently received accelerated FDA approval for MASH with significant to advanced fibrosis.^12^ In a phase 2, double-blind trial (NCT02970942) including 320 adults with biopsy-proven MASH and fibrosis F1–F3, aged 18–75 years, BMI > 25 kg/m^2^, with or without diabetes, the participants were randomized 3:3:3:1:1:1 to semaglutide 0.1 mg (*n* = 80), 0.2 mg (*n* = 78), 0.4 mg (*n* = 82), or placebo (*n* = 80) subcutaneously once daily for 72 weeks.^70^ Liver biopsies were obtained at baseline and week 72. MASH resolution without worsening of fibrosis occurred in 40% (0.1 mg dose), 36% (0.2 mg dose), 59% (0.4 mg dose), and 17% (placebo) of the participants (*p* < 0.001 for 0.4 mg vs. placebo). Fibrosis improvement of ≥1 stage without worsening of MASH was similar across the groups (49%, 32%, 43%, and 33%, respectively; *p* = 0.48 for 0.4 mg vs. placebo). The 0.4 mg dose led to 13% weight loss vs. 1% for placebo, with dose-dependent HbA1c reductions. Gastrointestinal side effects were most common at the highest dose compared with placebo (nausea: 42% vs. 11%; diarrhea: 20% vs. 14%; constipation: 22% vs. 12%; vomiting: 15% vs. 2%), causing only 4% discontinuation. The incidence of hepatic events was similar across the groups; gallbladder disorders were slightly more frequent with semaglutide than with placebo (6%, 5%, and 7% vs. 2%), with no cases of acute pancreatitis.

The ongoing ESSENCE trial (phase 3, double blind; NCT04822181) randomizes adults, aged ≥18 years, with biopsy-confirmed MASH and fibrosis F2–F3 to once-weekly semaglutide 2.4 mg subcutaneously or placebo for 240 weeks (*n* = 1,197). Primary endpoints include MASH resolution without worsening of fibrosis, fibrosis improvement without worsening of MASH, and cirrhosis-free survival. Interim analysis at week 72 in the first 800 participants (*n* = 534 semaglutide; *n* = 266 placebo) showed MASH resolution without worsening of fibrosis in 62.9% of the semaglutide group vs. 34.3% of the placebo group (*p* < 0.001), and fibrosis improvement without worsening of MASH in 36.8% of the semaglutide group vs. 22.4% of the placebo group (*p* < 0.001).^71^ Weight loss averaged 10.5% in the semaglutide group vs. 2% in the placebo group (*p* < 0.001), with concurrent improvements in HbA1c, C-reactive protein, lipid profile, and liver enzymes. VCTE decreased by 20%, ELF by 0.6 units, and N-terminal propeptide of type III collagen (Pro-C3) by 17% (*p* < 0.001). Gastrointestinal events were more frequent in the semaglutide group than in placebo group (nausea: 36.2% vs. 13.2%; diarrhea: 26.9% vs. 12.2%; constipation: 22.2% vs. 8.4%; vomiting: 18.6% vs. 5.6%), but only 13.4% were serious. In both groups, 3% of participants discontinued treatment due to adverse events. Gallbladder-related adverse events occurred in 2.5% of the semaglutide group vs. 1.5% of the placebo group; three (<1%) cases of acute pancreatitis were reported.

A separate phase 2 trial (NCT03987451) evaluated semaglutide in 71 adults, aged 18–75 years, with biopsy-confirmed MASH-related cirrhosis (BMI ≥ 27 kg/m^2^), randomized 2:1 to semaglutide 2.4 mg once-weekly subcutaneously (*n* = 47) or placebo (*n* = 24) for 48 weeks. The primary endpoint of ≥1 stage fibrosis improvement without worsening of MASH was not significantly different between the groups. Notably, no safety concerns arose.^72^

### GIP/GLP-1 receptor dual agonist

#### Tirzepatide

Tirzepatide, a weekly GIP/GLP-1 dual agonist, is approved for type 2 diabetes and obesity. The SYNERGY-NASH trial (phase 2, double blind; NCT04166773) included 190 adults with biopsy-confirmed MASH and fibrosis F2–F3, BMI 27–50 kg/m^2^, aged 18–80 years, with or without type 2 diabetes.^73^ The participants received tirzepatide 5 mg (*n* = 47), 10 mg (*n* = 47), 15 mg (*n* = 48), or placebo (*n* = 48) once weekly, subcutaneously for 52 weeks. Liver biopsies were performed at baseline and week 52. MASH resolution without worsening of fibrosis occurred in 44% (5 mg dose), 56% (10 mg dose), and 62% (15 mg) of participants in the tirzepatide group vs. 10% in the placebo group (*p* < 0.001). Fibrosis improvement of ≥1 stage without worsening of MASH occurred in 55% (5 mg dose), 51% (10 mg dose), and 51% (15 mg dose) of participants in the tirzepatide group vs. 30% in placebo group (*p* < 0.05). MRI-PDFF reduction ranged from 41.3% to 57% in the tirzepatide group vs. 9.8% in placebo group (*p* < 0.05), and cT1 improved by 70.7–107.2 ms (*p* < 0.05). VCTE decreased by 3.1–3.5 kPa (*p* < 0.05), ELF decreased by 0.45–0.50 units (*p* < 0.001), and Pro-C3 decreased by 40.1–45.7 μg/L (*p* < 0.001). Mean weight loss was 10.7%–15.6% in the tirzepatide group vs. 0.8% in the placebo group (*p* < 0.001). Gastrointestinal events were most common in the tirzepatide group compared with the placebo group (nausea: 44% vs. 12%; diarrhea: 27% vs. 23%; constipation: 15% vs. 6%; vomiting: 15% vs. 2%), mostly mild to moderate, with 4% discontinuation in both groups. Cirrhosis progression occurred in 3% of the tirzepatide group and 4% of the placebo group, with no cases of hepatic decompensation or drug-induced liver injury. Gallbladder-related adverse events occurred in 3% of the tirzepatide group vs. 2% of the placebo group, and no cases of acute pancreatitis were reported.

The ongoing SYNERGY-OUTCOMES trial (phase 3, double-blind; NCT07165028) randomizes adults, aged ≥18 years, with MASLD and fibrosis F2–F3 based on non-invasive tests (liver fat content ≥ 8%, ELF scores ≥ 9 and ≤10.8, and VCTE-LSM ≥ 10 and <20 kPa) to receive once-weekly subcutaneous tirzepatide, retatrutide, or placebo for approximately 224 weeks (*n* = 4,500). The primary endpoint is time to first occurrence of a composite major adverse liver outcomes (MALO), including progression to cirrhosis, development of hepatic decompensation, liver transplantation, or all-cause mortality. Estimated study completion is Q3 2032.

### Glucagon/GLP-1 receptor dual agonist

#### Pemvidutide

Pemvidutide is a weekly glucagon/GLP-1 receptor dual agonist under development for MASH and obesity. The IMPACT trial (phase 2, double blind, dose-finding; NCT05989711) enrolled 212 adults, aged 18–75 years, with biopsy-confirmed MASH and fibrosis F2–F3, BMI ≥ 27 kg/m^2^, randomized 1:2:2 to pemvidutide 1.2 mg, 1.8 mg, or placebo once weekly, subcutaneously, for 24 weeks.^74^ MASH resolution without worsening of fibrosis occurred in 58% (1.2 mg dose) and 52% (1.8 mg dose) of the pemvidutide group vs. 20% in placebo group (*p* < 0.001). ≥1 stage fibrosis improvement without worsening of MASH occurred in 33% (1.2 mg dose) and 36% (1.8 mg dose) of the pemvidutide group vs. 28% in placebo group. A ≥30% reduction in MRI-PDFF was observed in 82%–84% of the pemvidutide group vs. 20% in the placebo group, and a ≥80 ms reduction in cT1 was observed in 74%–78% of the pemvidutide group versus 28% in the placebo group. Liver stiffness decreased by 2.2–3.7 kPa, ELF decreased by 0.5–0.6 units, and Pro-C3 decreased by 14.7–15.3 μg/L. Body weight decreased by 4.8%–5.8% with pemvidutide compared with a 0.5% decrease with placebo. Gastrointestinal events were most common in the pemvidutide group compared with the placebo group (nausea: 41% vs. 14%; diarrhea: 21% vs. 8%; constipation: 13% vs. 9%), mostly mild to moderate in severity. Discontinuation occurred in 1% of the pemvidutide group vs. 2% of the placebo group. Serious adverse events occurred in 3% of participants across the pemvidutide and placebo groups, with none considered treatment related.

#### Survodutide

Survodutide is a weekly glucagon/GLP-1 receptor dual agonist with more potent activity on the GLP-1 receptor (∼8-fold higher) compared with its glucagon receptor.^75^ Phase 2, double-blind, dose-finding trial (NCT04771273) enrolled 293 adults with biopsy-proven MASH, fibrosis F1–F3, BMI ≥ 25 kg/m^2^, aged 18–80 years, with or without diabetes.^68^ The participants were randomized 1:1:1:1 to survodutide 2.4 mg (*n* = 73), 4.8 mg (*n* = 72), 6 mg (*n* = 74), or placebo (*n* = 74) once weekly, subcutaneously, for 48 weeks.^68^ MASH improvement without worsening of fibrosis occurred in 47% (2.4 mg dose), 62% (4.8 mg dose), and 43% (6 mg dose) of the survodutide group vs. 14% in the placebo group (*p* < 0.001). ≥1 stage fibrosis improvement occurred in 34% (2.4 mg dose), 36% (4.8 mg dose), and 34% (6 mg dose) of the survodutide group vs. 22% in the placebo group. A ≥30% reduction in MRI-PDFF was observed in 57%–67% of the survodutide group vs. 14% in the placebo group. Absolute weight loss was 10.2–13.1 kg in the survodutide group vs. 0.6 kg in the placebo group. HbA1c also decreased by up to 0.8% in the survodutide group. Gastrointestinal events were more frequent in the survodutide group than in the placebo group (nausea: 66% vs. 23%; diarrhea: 49% vs. 23%; constipation: 21% vs. 15%; vomiting: 41% vs. 4%), leading to discontinuation in 16% of participants in the survodutide group vs. 1.4% in the placebo group.

The ongoing LIVERAGE trial (phase 3, double-blind; NCT06632444) randomizes adults, aged ≥18 years, with biopsy-confirmed MASH and fibrosis F2–F3 to receive once-weekly survodutide, reaching a maximum dose of 6 mg subcutaneously, or placebo for approximately 7 years (*n* = 1,800). LIVERAGE consists of two parts. Primary endpoints of part 1 (after 52 weeks of treatment) include MASH resolution without worsening of fibrosis and at least a 1-point improvement in fibrosis without worsening of MASH. The primary endpoint of part 2, which will continue for approximately 7 years, is time to first occurrence of liver-related events or all-cause mortality. Estimated study completion is Q4 2031.

### Glucagon/GIP/GLP-1 receptor triple agonist

#### Efocipegtrutide

Efocipegtrutide is a weekly glucagon/GIP/GLP-1 triple agonist under investigation for MASH. The HM-TRIA-201 trial (phase 2, double blind; NCT04505436) includes adults with biopsy-confirmed MASH, fibrosis F1–F3, MRI-PDFF ≥ 8%, with or without diabetes, randomized 1:1:1:1 to 2 mg, 4 mg, 6 mg, or placebo once weekly for 52 weeks (*N* ≈ 240). The primary endpoint is MASH resolution without worsening of fibrosis. Estimated study completion is Q3 2026.

### Clinical insights and translational relevance

Collectively, the effects of incretin-based therapies in MASH can be interpreted through a unifying framework in which each receptor agonist targets distinct but complementary pathogenic nodes driving disease progression.^76^ Across trials, incretin-based therapies consistently demonstrate meaningful reductions in the liver fat content (as assessed by histology or imaging) and inflammatory activity (as assessed by histology or liver enzymes), as well as weight loss and improvements in glycemic control and lipid profiles.^10^^,^^13^^,^^22^^,^^68^^,^^69^^,^^70^^,^^71^^,^^73^^,^^74^^,^^77^^,^^78^^,^^79^^,^^80^^,^^81^^,^^82^^,^^83^^,^^84^^,^^85^ Within this context, a recent systematic review and network meta-analysis of 29 randomized controlled trials involving 9,324 participants with biopsy-proven MASH further supports the overall efficacy of incretin-based therapies, with tirzepatide and survodutide ranking among the top interventions for MASH resolution without worsening fibrosis based on surface under the cumulative ranking curve (SUCRA) analyses.^15^ However, these findings do not yet incorporate phase 3 ESSENCE trial data, which demonstrated comparable therapeutic efficacy with semaglutide. Notably, in addition to their hepatic and metabolic benefits, GLP-1 receptor agonists have demonstrated significant cardiovascular and renal protective effects.^86^^,^^87^^,^^88^

In contrast, fibrosis improvement is less consistently observed across all classes and lags behind MASH resolution.^13^^,^^68^^,^^69^^,^^70^^,^^71^^,^^73^^,^^74^^,^^78^ This temporal dissociation likely reflects the fundamental differences in disease biology—hepatic steatosis and inflammatory activity are dynamic and relatively reversible processes, whereas fibrosis represents a structurally entrenched wound-healing response characterized by persistent hepatic stellate cell activation and slow extracellular matrix turnover.^3^^,^^89^ In this scenario, steatosis reduction appears to be the earliest and most rapidly responsive therapeutic outcome, driven primarily by systemic weight loss and restoration of free fatty acid flux between adipose tissue and the liver.^90^^,^^91^^,^^92^^,^^93^ As hepatic lipid accumulation decreases, steatohepatitis improves through reduced hepatocellular stress and lobular inflammation, alongside both weight-dependent mechanisms and the immunomodulatory effects of incretin signaling.^22^^,^^25^^,^^94^ In contrast, fibrosis regression remains delayed across pharmacological classes, as it requires the deactivation of hepatic stellate cells and sustained extracellular matrix remodeling.^95^^,^^96^^,^^97^ Consequently, even in the presence of substantial metabolic and inflammatory improvement, histological fibrosis resolution may require longer treatment durations than have been captured in most clinical trials. Within this framework, semaglutide, tirzepatide, and survodutide appear to show greater antifibrotic effects.^15^^,^^68^^,^^71^^,^^73^ However, larger head-to-head and phase 3 trials are needed to confirm the relative efficacy across classes.

Additionally, differences in the magnitude of hepatic responses across drug classes reflect their distinct but complementary mechanisms of action. GLP-1 receptor agonists primarily exert their hepatic benefits through central and peripheral mechanisms that reduce energy intake and promote weight loss, thereby decreasing adipose tissue lipolysis and the flux of free fatty acids to the liver.^2^^,^^7^ In addition to these weight-dependent effects, preclinical and mechanistic evidence supports additional weight-independent actions on hepatic outcomes mediated through the direct modulation of inflammatory and fibrotic pathways.^22^^,^^23^ GIP/GLP-1 dual agonist may further extend this framework by also acting on white adipose tissue biology, as suggested by preclinical studies demonstrating enhanced lipid-buffering and lipid-storage capacities.^49^ The consequent reduction in the ectopic lipid spillover may contribute to the greater magnitude of hepatic fat reduction and weight loss observed with GIP/GLP-1 dual agonism compared with GLP-1 agonism. By comparison, dual or triple agonists incorporating glucagon receptor agonism introduce a distinct, liver-centric mechanistic dimension by directly stimulating hepatic fatty acid oxidation and promoting a fasting-like metabolic state, thereby increasing intrahepatic lipid turnover and energy expenditure.^2^^,^^13^^,^^57^ When combined with GLP-1 or GIP receptor agonism, this additional direct, liver-centric effect of glucagon receptor agonism may contribute to greater reductions in hepatic steatosis and inflammation, as well as enhanced weight loss and metabolic improvements. However, further studies in humans are needed to firmly elucidate the contribution of GIP and glucagon signaling and the determinants of fibrosis response.

Safety profiles across these drug classes are broadly consistent with their mechanisms of action. Mild-to-moderate gastrointestinal adverse events, including nausea, vomiting, diarrhea, and constipation, predominate across GLP-1-based regimens and appear to correlate with higher doses and transient treatment discontinuation. Importantly, no signal of intrinsic hepatotoxicity has emerged across trials.^2^ Overall, the glucagon-containing therapy survodutide appears to be associated with a greater burden of gastrointestinal adverse events and higher rates of treatment discontinuation.^10^^,^^68^ Consistent with these findings, evidence from real-world studies and prescription/renewal analyses suggests relatively high discontinuation rates with GLP-1-based therapies over time, reaching approximately 30%–50% within the first year, likely driven by gastrointestinal tolerability, which highlights the importance of patient education, long-term support, and adherence strategies in clinical practice.^98^^,^^99^ Notably, a key issue that remains incompletely understood is the extent of muscle loss associated with weight reduction observed with GLP-1 receptor agonists, and whether mono, dual, and triple agonists exhibit differential effects on the relative proportions of adipose versus muscle tissue loss.^22^^,^^100^^,^^101^ This may be particularly relevant in patients with cirrhosis, in whom sarcopenia is a major prognostic determinant and associated with an increased risk of hepatic decompensation.^102^^,^^103^^,^^104^ Accordingly, careful assessment and monitoring of body composition are warranted in patients with established cirrhosis receiving these agents.

## Conclusion and future directions

GLP-1 receptor mono, dual, and triple agonists represent some of the most promising pharmacotherapies in the evolving landscape of treatments for MASH-related fibrosis. Across all incretin-based therapies, weight loss remains a central mediator of hepatic improvement through reductions in adipose tissue mass, systemic insulin resistance, and circulating lipotoxic substrates. However, emerging evidence suggests that these agents also exert effects that extend beyond weight reduction. GLP-1 receptor agonists may directly attenuate the inflammatory signaling pathways, GIP receptor activation may improve adipose tissue lipid handling independently of weight loss, and glucagon receptor agonism may enhance hepatic oxidative metabolism irrespective of changes in body mass. The clinical effects observed in trials, therefore, likely reflect a composite of weight-mediated and weight-independent systemic and liver-centric benefits. Their effects on MASH follow a predictable temporal sequence in which improvements in steatosis and steatohepatitis precede and exceed changes in fibrosis, reflecting fundamental differences in disease biology. Consequently, fibrosis outcomes may require longer follow-ups in clinical trials to fully reflect structural liver remodeling. The ongoing phase 3 trials will be pivotal in defining their clinical benefit and clarifying their impact on long-term outcomes. Nevertheless, several critical questions remain unresolved, including the optimal duration of therapy, challenges with adherence due to gastrointestinal side effects, and the sources of heterogeneity in treatment response, as well as the contribution of GIP and glucagon signaling in humans and the determinants of fibrosis response. Importantly, it remains unclear whether incretin-based therapies can reverse cirrhosis, and whether their use would have a beneficial or detrimental effect given the potential for significant muscle loss with continued use in the setting of cirrhosis. Further studies are needed to characterize the impact of these agents on skeletal muscle mass in patients with MASH and to determine whether mono, dual, and triple agonists have differential effects on muscle outcomes in advanced liver disease. Another unresolved issue is whether these agents confer a fibrosis benefit in established cirrhosis, a population currently excluded from ongoing phase 3 trials. Addressing these questions is essential to fully establish their role in the management of advanced MASH.

## Acknowledgments

R.L. receives funding support from 10.13039/100006108NCATS (5UL1TR001442), 10.13039/100000062NIDDK (P30DK120515), and John C Martin Foundation (RP124).

## Author contributions

All authors contributed to the drafting and revision of this manuscript.

## Declaration of interests

R.L. serves as a consultant to Aardvark Therapeutics, Altimmune, Arrowhead Pharmaceuticals, AstraZeneca, Cascade Pharmaceuticals, Eli Lilly, Gilead, Glympse Bio, Inipharma, Intercept, Inventiva, Ionis, Janssen Inc., Lipidio, Madrigal, Neurobo, Novo Nordisk, Merck, Pfizer, Sagimet, 89 bio, Takeda, Terns Pharmaceuticals, and Viking Therapeutics. In addition, his institution received research grants from Arrowhead Pharmaceuticals, AstraZeneca, Boehringer-Ingelheim, Bristol-Myers Squibb, Eli Lilly, Galectin Therapeutics, Gilead, Intercept, Hanmi, Intercept, Inventiva, Ionis, Janssen, Madrigal Pharmaceuticals, Merck, Novo Nordisk, Pfizer, Sonic Incytes, and Terns Pharmaceuticals. R.L. is also a co-founder of LipoNexus Inc.
